# Non-Targeted Metabolomics Analysis Reveals Metabolite Profiles Change During Whey Fermentation with *Kluyveromyces marxianus*

**DOI:** 10.3390/metabo14120694

**Published:** 2024-12-09

**Authors:** Yansong Gao, Lei Gao, You Kang, Ge Yang, Zijian Zhao, Yujuan Zhao, Shengyu Li

**Affiliations:** 1 Institute of Agro-Food Technology, Jilin Academy of Agricultural Sciences (Northeast Agricultural Research Center of China), Changchun 130033, China; gysgerry@126.com (Y.G.); redhuman@126.com (L.G.); kangkang.1982@163.com (Y.K.); yangge1900@163.com (G.Y.); jj275093139@foxmail.com (Z.Z.); zhaojaas@163.com (Y.Z.); 2 National R&D Center for Milk Processing, Changchun 130033, China

**Keywords:** whey, *Kluyveromyces marxianus*, non-targeted metabolomics, metabolic pathways, differential metabolites

## Abstract

**Background:** Whey fermentation could produce bioactive substances with immunomodulatory effects, metabolic syndrome modulation, and antioxidant properties, thereby imparting functional characteristics to products and facilitating the development of novel foods with health-promoting potential. **Methods:** A non-targeted metabolomics approach using liquid chromatography–mass spectrometry (LC-MS) was employed to investigate changes in the metabolite profiles of whey fermented by *Kluyveromyces marxianus* strain KM812 over varying fermentation durations. **Results:** The findings demonstrated a progressive enrichment of metabolites over time. A total of 151 differential metabolites were identified and categorized primarily into amino acids, peptides, and analogues, fatty acids and conjugates, and carbohydrates and conjugates, as well as benzoic acids and derivatives. The highest relative content of whey metabolites was observed at 48 h of fermentation, with a cumulative increase of 1.45-fold, 1.49-fold, 3.39-fold, and 1.24-fold for peptides and amino acids, peptides, and analogues, fatty acids and conjugates, and carbohydrates and conjugates, respectively. Kyoto Encyclopedia of Genes and Genomes (KEGG) analysis revealed associations with 23 specific metabolites and delineated 9 metabolic pathways, predominantly involved in amino acid and lipid metabolism. **Conclusions:** Based on the above, KM812 could effectively degrade macromolecular substances in whey into small molecules such as L-isoleucine, ornithine, betaine, α-linolenic acid, and palmitoleic acid, thereby influencing the nutritional and functional properties of whey. In-depth analysis of the metabolic products in KM812-fermented whey could provide a theoretical basis for the development of functional foods derived from small molecules in the future.

## 1. Introduction

Whey constitutes a significant by-product of cheese or casein production, typically representing 85–95% of milk by volume [1]. Commonly processed into products such as whey protein concentrate, whey protein isolate, whey powder, and lactose, whey plays a pivotal role in the food industry [2,3]. Utilizing whey effectively is crucial not only for transforming it into value-added commodities but also for minimizing waste and potential environmental impact. Microbial fermentation stands out as a vital method for comprehensive whey utilization, enhancing its economic value. During fermentation, whey generates bioactive peptides, organic acids, and various physiologically active compounds, known for their immunomodulatory [4], hypotensive [5], antioxidant [6], and antimicrobial [7] properties. Microorganisms commonly employed in whey fermentation include *Bifidobacterium bifidum*, *Lactobacillus acidophilus*, *Lactobacillus plantarum*, *Lactobacillus rhamnosus*, and yeasts. These microorganisms not only influence the texture, taste, and flavour of fermented whey products, they also impart functional attributes [8]. For instance, whey fermented by *Bifidobacteria* could generate immunoregulatory peptides, denatured hypoallergenic α-LA and β-LG, thereby upregulating intestinal probiotics and intestinal metabolites (SCFAs), regulating inflammatory factors, and downregulating the levels of allergy markers. *L. acidophilus* fermented whey could produce antioxidant peptides, phenols, flavonoids, and it could also increase the activity of SOD, GSH, and GPx, thereby enhancing the ability to scavenge free radicals and reducing the production of melanin. The fermented whey could be manufactured into fermented dairy products, bread, fermented meat products, fermented whey beverages, whey spirits, whey vinegars, etc. Nevertheless, challenges exist in the application of microorganisms for whey fermentation. Variations in fermentation outcomes among different microbial species, and even different strains within the same genus, underscore the necessity of strain-specific screening for optimal whey fermentation processes.

*Kluyveromyces marxianus* is a class of microorganisms generally recognised as safe (GRAS) for consumption [9]. This yeast species is distinguished by its robust industrial potential, characterized by high heat resistance and rapid growth rates [10]. *K. marxianus* is particularly noted for its efficient lactose catabolism, a trait extensively explored in whey fermentation, notably for ethanol production [11,12,13]. In food processing, *K. marxianus* has been instrumental in developing products such as whey wine and whey-based beverages. For instance, Castillo et al. employed *K. marxianus* and *Debaryomyces hansenii* in monoculture and co-culture to ferment cheese whey, producing aromatic heterologous wines [14]. Similarly, Rivera Flores et al. utilized *Saccharomyces cerevisiae*, *K. marxianus*, and *Brettanomyces claussenii* to ferment acid whey, creating distinctively flavoured beverages [15]. *K. marxianus* has demonstrated significant utility in whey fermentation, enhancing nutritional profiles, processing characteristics, and facilitating the production of specialized fermented products [16,17].

Our laboratory had isolated and identified a new strain of *Kluyveromyces marxianus* from Tibetan lingzhi mushrooms, which had been utilized for the fermentation of cheese whey. Using liquid chromatography–mass spectrometry (LC-MS) and multivariate statistical analysis, we conducted a non-targeted metabolomics study to explore the metabolic pathways of whey at different fermentation times. This research aims to investigate the dynamic changes in chemical components during the fermentation process and analyze the bioactive constituents involved. This research strategy is intended to provide insights into the application of *K. marxianus* in whey fermentation, laying a theoretical foundation for the subsequent development of small-molecule functional foods. This study has significant practical implications for the transformation of whey into value-added products, reducing whey waste, and mitigating potential pollution, while also offering a new perspective for the effective utilization of whey.

## 2. Materials and Methods

### 2.1. Chemicals and Materials

The whey was a by-product of the processing of Halloumi cheese in our laboratory, containing 0.85% protein and 0.53% fat, pH = 6.6. LC-MS-grade acetonitrile (ACN) and methanol (MeOH) were purchased from Fisher Scientific (Loughborough, UK). Formic acid was obtained from TCI (Shanghai, China). Ammonium formate was obtained from Sigma-Aldrich (Shanghai, China). 2-Amino-3-(2-chloro-phenyl)-propionic acid was obtained from Aladdin (Shanghai, China). Ultrapure water was generated using a Milli-Q system (Millipore, Bedford, MA, USA). Additional chemicals that were utilized in this study were all of analytical or chromatographic grade.

### 2.2. Bacteria, Culture Media, and Growth Condition

*K. marxianus* KM812 was isolated from Tibetan mushroom, and the strain was conserved in the Chinese Centre for Conservation of Typical Cultures (Conservation No. CCTCC NO:M 20221366). *K. marxianus* KM812 was cultured in YPD liquid medium (1.0% yeast extract, 2.0% polypeptone and 2.0% glucose) at 30 °C for 48 h. The precipitate was collected by centrifugation at 5000 r/min for 10 min at 4 °C and resuspended in sterile saline (0.9% sodium chloride). The bacterial count in the suspension was equal to 1.0 × 10^10^ CFU/mL.

### 2.3. Sample Preparation

The whey was pasteurized (70 °C, 20 min), cooled to 30 °C, inoculated with 5% *K. marxianus* KM812, and left to ferment at 30 °C for 72 h. Fermented whey samples were collected at 0, 24, 48, and 72 h. The samples were set up in six replicates at each time point. The fermentation broth was lyophilised and 30 mg was extracted with 500 µL of methanol, centrifuged (12,000 r/min, 4 °C, 10 min), and the methanol was recovered under reduced pressure. The sample was re-dissolved by adding 150 µL of 2-chloro-L-phenylalanine solution (4 mg/L) prepared in 80% methanol in water, and the supernatant was filtered through a 0.22 μm microporous membrane for LC-MS detection [18].

### 2.4. LC-MC Analysis

Liquid chromatography conditions: The parameters of Thermo Vanquish (Thermo Fisher Scientific, Waltham, MA, USA) UHPLC system were set as follows. The chromatographic column was ACQUITY UPLC^®^HSS T3 (2.1 × 150 mm, 1.8 µm) (Waters, Milford, MA, USA), the temperature of the column was 40 °C, the injection volume was 2 μL, and the flow rate was 0.25 mL/min. The column temperature was 40 °C, the injection volume was 2 μL, and the flow rate was 0.25 mL/min. The mobile phase in positive-ion mode was a mixture of 0.1% formic acid acetonitrile (B2) and 0.1% formic acid water (A2), and the gradient elution was as follows: 0–1 min, 98% A2; 1–9 min, 98%~50% A2; 9–12 min, 50%~2% A2; 12–13.5 min, 2% A2; 13.5~14 min, 2%~98% A2; and 13.5~14 min, 2%~98% A2. ~14 min, 2%~98% A2; 14~20 min, 98% A2. The mobile phase in negative-ion mode was a mixture of acetonitrile (B3) and 5 mM ammonium formate in water (A3), and the gradient elution as follows: 0~1 min, 98% A3; 1~9 min, 98%~50% A3; 9~12 min, 50%~2% A3; 12~13.5 min, 2% A3; 13.5–14 min, 2%~98% A3; 14–17 min, 98% A3 [19].

Mass spectrum conditions: Thermo Q Exactive Focus mass spectrometry detector (Thermo Fisher Scientific, USA) parameter settings: spray voltage of 3.50 kV, −2.50 kV, positive and negative modes; sheath gas and auxiliary gas of 30 arb and 10 arb, respectively; capillary temperature of 325 °C; resolution of 70,000, scanning range 100~1000 *m*/*z*; HCD was subjected to secondary cleavage with a secondary resolution of 17,500, collision energy of 30 eV, 3 ions were fragmented before the acquisition of the signal, and dynamic exclusion was used to remove unnecessary MS/MS information [20].

### 2.5. Data Processing and Multivariate Analysis

The original mass spectrometry raw files were converted to an mzXML format using the MSConvert tool from the Proteowizard software package (v3.0.8789). Peak detection, filtering, and alignment were performed with the R XCMS package, resulting in a substance quantification list. The parameter settings included bw = 2, ppm = 15, peakwidth = c(5, 30), mzwid = 0.015, mzdiff = 0.01, and method = “centWave”. For substance identification, public databases such as HMDB, MassBank, LipidMaps, mzCloud, and KEGG, as well as a self-constructed substance library, were utilized, with a parameter setting of 30 ppm. A LOESS signal correction method based on QC samples was employed for data correction to eliminate systematic errors. During data quality control, substances with an RSD greater than 30% in the QC samples were filtered out.

The R package Ropls was used for Principal Components Analysis (PCA), Partial Least Squares-Discriminant Analysis (PLS-DA), and Orthogonal Partial Least Squares Discriminant Analysis (Orthogonal Projections to Latent Structures Discriminant Analysis, OPLS-DA) dimensionality reduction analyses. Differential metabolite screening was performed based on the *p*-value <0.05 calculated by statistical testing and Variable Importance in the Projection (VIP) >1 calculated by the OPLS-DA dimensionality reduction method. Functional pathway enrichment and topological analysis of the screened differential metabolite molecules were performed using the MetaboAnalyst software package, and metabolic pathway analysis was performed by referring to the Kyoto Encyclopedia of Genes and Genomes (KEGG) pathway database.

## 3. Results

### 3.1. Metabolic Profiling of Different Stages of K. Marxianus KM812-Fermented Whey

**Figure 1:**
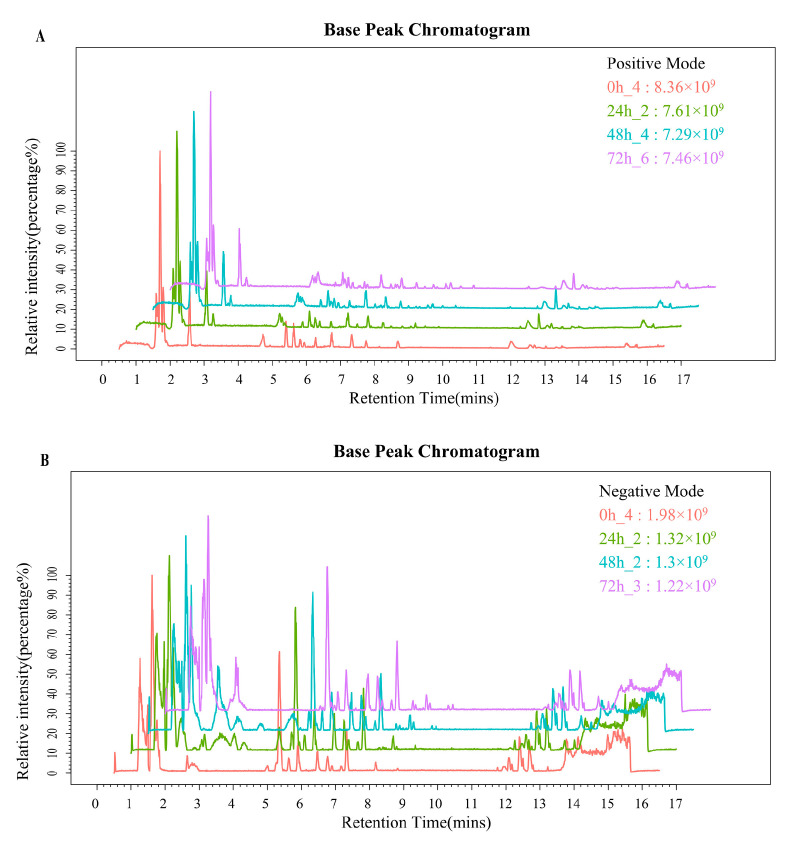
(**A**,**B**) PBC of ESI+ and ESI− mode in whey at different fermentation times.

A Base Peak Chromatogram (BPC) was extracted for LC-MS analysis of whey samples collected at the 0 h, 24 h, 48 h, and 72 h fermentation time points (Figure 1). According to the BPC, there were differences in the metabolic profiles among samples from different time points. The intensities of spectral signals demonstrated a clear time-dependent trend, indicating substantial increases in secondary metabolite levels during fermentation. A comprehensive total of 7995 metabolites were identified in the fermented whey samples, with 4909 metabolites detected in ESI+ mode (Figure 1A) and 3086 in ESI- mode (Figure 1B).

### 3.2. Trend Analysis of Changes in Different Stages of K. marxianus KM812-Fermented Whey

**Figure 2:**
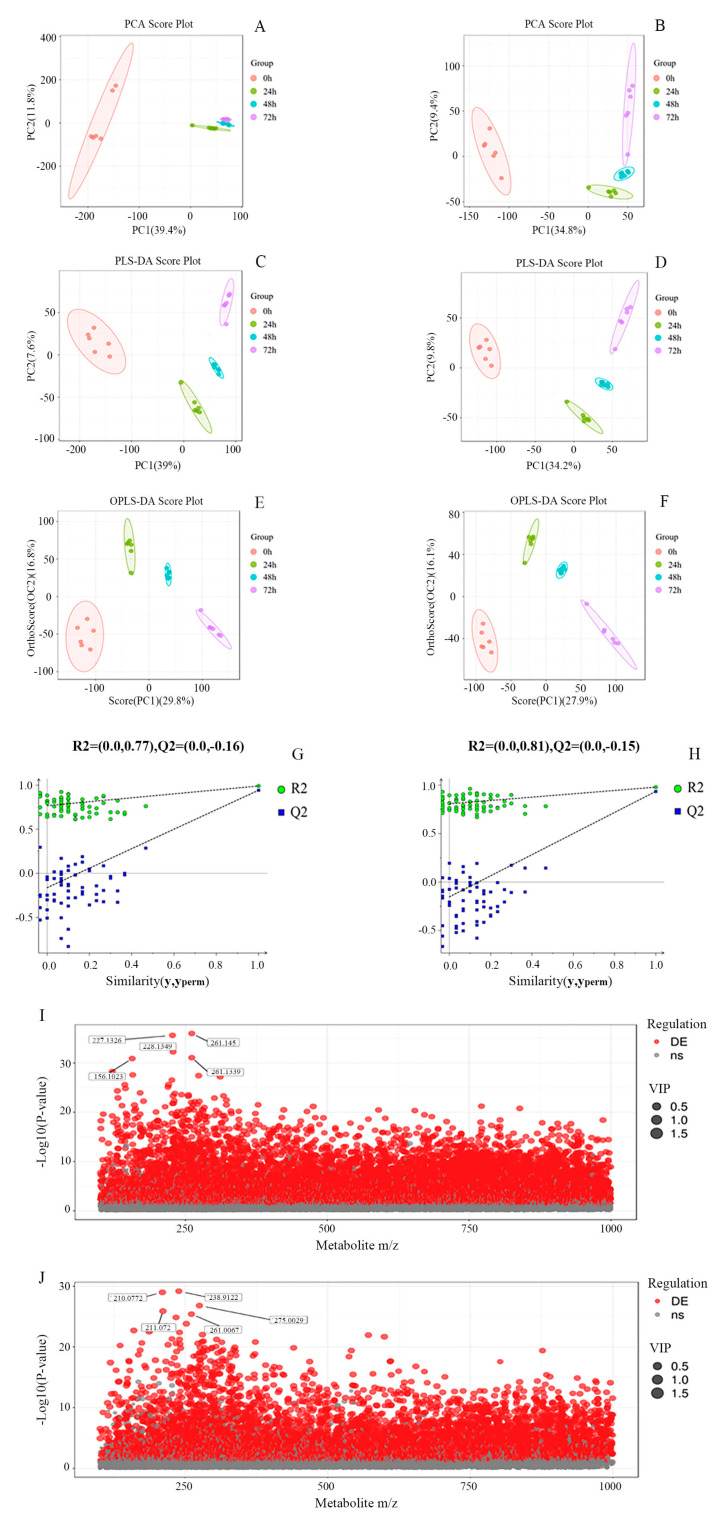
(**A**−**J**) PCA score plots (**A**,**B**), PLS−DA score plots (**C**,**D**), OPLS−DA score plots (**E**,**F**), accompanied by permutation tests (**G**,**H**), and charge ratio and *p* value scatter plots (**I**,**J**) of whey at different fermentation times in the ESI + mode and in the ESI− mode, respectively (each point in (**I**,**J**) denotes one metabolite, with the horizontal coordinates denoting the mass−to−charge ratios of the tertiary substances; the vertical coordinates denoting the *p*−values of the −log10 of the *p* value. Larger values of the vertical coordinate indicate more significant differential expression. Red dots represent up−regulated differentially expressed metabolites, grey dots represent metabolites that did not satisfy the filtering parameters, and the size of the dots indicates the VIP value).

Image recognition methods, coupled with PCA and PLS-DA, were employed to assess metabolite aggregation, disaggregation, and intra- and intergroup variations. The PCA score plots (Figure 2A,B) illustrate that the principal component contributions were 46.2% in the ESI+ mode and 44.2% in the ESI- mode. A noticeable trend of within-group sample aggregation and between-group sample separation was observed, underscoring the robustness and reproducibility of the analysis. The validity of the model was further confirmed using PLS-DA (Figure 2C,D), demonstrating similar changes as observed in PCA, thereby affirming the model’s robust fit and high predictive capacity.

To mitigate overfitting and minimize systematic noise unrelated to cluster separation, OPLS-DA was employed (Figure 2E,F) to identify potential markers. Additionally, a random permutation test (*n* = 100, Figure 2G,H) was conducted to assess differences in metabolites during fermentation. The results depicted in Figure 2G,H show that all blue Q2 points were consistently lower than the corresponding original blue Q2 points located on the extreme right, indicating robustness, reproducibility, and predictive capability of the model.

Based on the results of OPLS-DA, metabolites meeting the criteria of a *p*-value less than 0.05 and a VIP score greater than 1.0 were selected. These criteria were applied in conjunction with scatter plots depicting metabolite ratios versus *p*-values (Figure 2I,J) to illustrate the distribution of differential substances in fermented whey. A total of 151 differential metabolites were identified in the whey fermentation broth, indicating significant alterations in whey metabolites due to *K. marxianus* KM812 fermentation.

### 3.3. Differential Metabolites Analysis of K. marxianus KM812-Fermented Whey

To further analyze the differential metabolites of *K. marxianus* KM812-fermented whey, 151 metabolites were classified into 29 categories (Table S1). These metabolites predominantly belong to four groups: derivatives of amino acids, peptides, and analogues; fatty acids and conjugates; carbohydrates and carbohydrate conjugates; and benzoic acids and derivatives.

**Figure 3:**
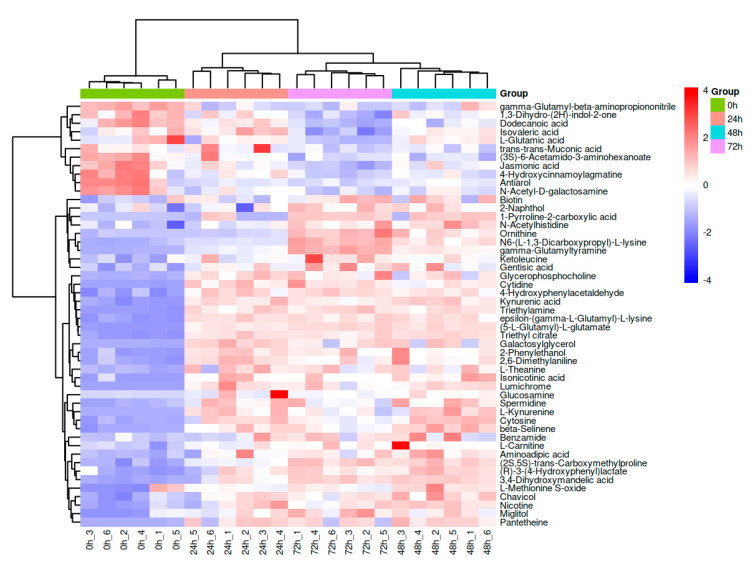
Hierarchical clustering heatmap of differential metabolites of fermented whey at different stages of fermentation (the magnitude of the relative content in the plot is shown by differences in colour, with redder colours showing higher expression and bluer colours showing lower expression).

To provide a more intuitive description of the differential metabolites in *K. marxianus* KM812-fermented whey, an agglomerative hierarchical clustering heat map (Figure 3) was employed to visualize the specific changes in metabolites across different fermentation stages. The clustering dendrogram above the heat map reveals two distinct branches: the initial branch at 0 h, and the subsequent branch encompassing 24, 48, and 72 h, displaying noticeable colour variations between them. Notably, the samples at 24, 48, and 72 h exhibit similar colour patterns, indicating comparable metabolite relative content. The clustering of the 48 h and 72 h samples further suggests similarity in metabolite expression patterns, signifying consistent trends in their changes. The trend of the colours in Figure 3 showed that some of the differential metabolites gradually accumulated with time and some of them gradually decreased with time.

**Figure 4:**
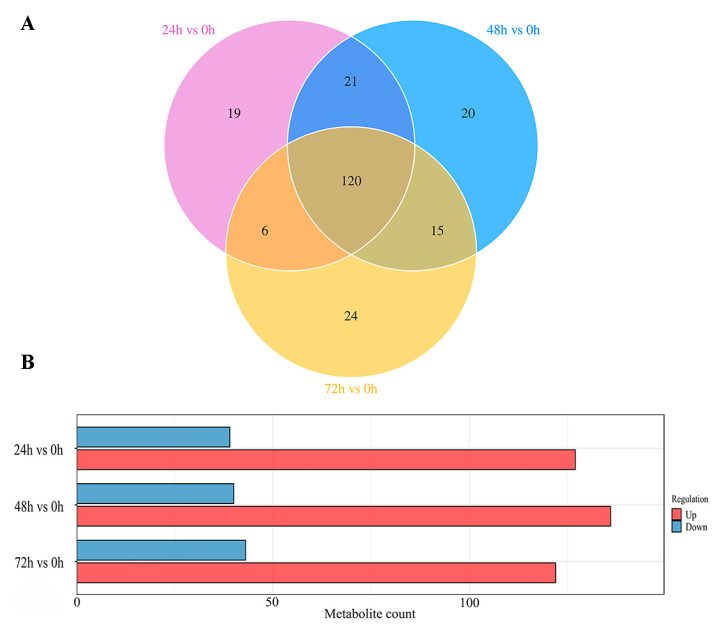
(**A**,**B**) Venn diagram of differential metabolites in intergroup comparisons (**A**); histogram of differential metabolite statistics for group comparisons (**B**) (X−axis indicates number of differential metabolites and Y−axis indicates group comparison conditions).

The Venn diagram (Figure 4A) illustrates that a total of 120 differential metabolites were shared among the 24 h versus 0 h, 48 h versus 0 h, and 72 h versus 0 h comparison groups, with 19, 20, and 24 differential metabolites being unique to each respective comparison group, respectively. In addition, the statistical analysis of intergroup differences in differential metabolites (Figure 4B) revealed that, in the 24 h versus 0 h comparison group, 127 metabolites were upregulated and 39 downregulated; in the 48 h versus 0 h comparison group, 136 metabolites were upregulated and 40 downregulated; and in the 72 h versus 0 h comparison group, 122 metabolites were upregulated and 43 downregulated. These results indicate that the 48 h samples exhibited the largest number of differential metabolites. It was also demonstrated that the differential metabolites changed with increasing time, in agreement with the results of Figure 3.

**Figure 5:**
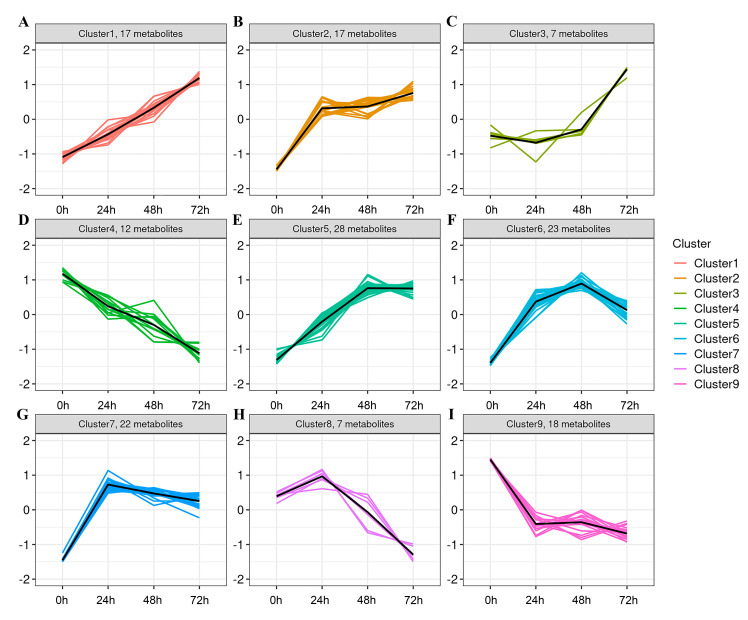
(**A**–**I**) Molecular expression pattern plot (each subplot represents a different cluster, with the x−axis indicating fermentation time and the y−axis representing the average expression level of the molecule within each group. Cluster1 Includes 17 metabolites (**A**); Cluster2 Includes 17 metabolites (**B**); Cluster3 Includes 7 metabolites (**C**); Cluster4 Includes 12 metabolites (**D**); Cluster5 Includes 28 metabolites (**E**); Cluster6 Includes 23 metabolites (**F**); Cluster7 Includes 22 metabolites (**G**); Cluster8 Includes 7 metabolites (**H**); Cluster1 Includes 18 metabolites (**I**);).

For a more accurate analysis of trends in differential metabolite changes, K-means clustering analysis (Figure 5, Table 1) was conducted to better elucidate the trends in differential metabolite variation during whey fermentation. Based on similar patterns of change, the metabolites were classified into nine clusters. Clusters 1, 2, 3, and 5 exhibited a significant increase with the extension of fermentation time. Specifically, Cluster 1 comprises 17 differential metabolites (Figure 5A), Cluster 2 comprises 17 differential metabolites (Figure 5B), Cluster 3 comprises 7 differential metabolites (Figure 5C), and Cluster 5 comprises 28 differential metabolites (Figure 5E). Clusters 4 and 9 showed a decreasing trend with increasing fermentation time, containing 12 and 18 differential metabolites, respectively (Figure 5D,I). Other clusters demonstrated an initial increase followed by a decrease. The metabolites that significantly increased over time in these clusters were primarily amino acids, peptides, and analogues, fatty acids and conjugates, carbohydrates and conjugates, and benzoic acids and derivatives. These results suggest that *K. marxianus* KM812 fermentation effectively degrades macromolecular substances, resulting in the accumulation of small molecule compounds within the fermented whey. These compounds encompass L-glutamic acid, L-isoleucine, and ornithine from peptides and amino acids; alpha-linolenic acid, linoleic acid, palmitoleic acid, and jasmonic acid from lipids; D-ribose and mannitol from carbohydrates and carbohydrate conjugates; and (R)-3-(4-hydroxyphenyl)lactate, 4-hydroxyphenylacetaldehyde, and gentisic acid from benzoic acids and derivatives. These findings underscore the enhancement of whey digestion and absorption, along with the enrichment of nutritional and functional components through KM812 fermentation.

### 3.4. Metabolic Pathway Analysis of K. marxianus KM812-Fermented Whey

**Figure 6:**
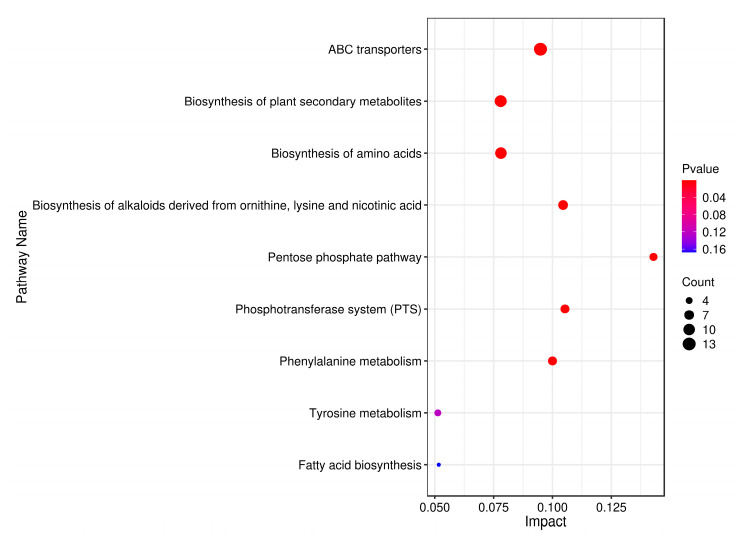
Metabolic pathway impact factor bubble diagram. (Each point in the diagram represents a metabolic pathway, the horizontal coordinates are the Impact values enriched into different metabolic pathways, and the vertical coordinates are the enriched pathways. The dots indicate the corresponding number of metabolic molecules in the pathway. The colours correlate with the *p*-value; the redder the colour, the smaller the *p*-value, and the bluer the colour, the larger the *p*-value.)

**Figure 7:**
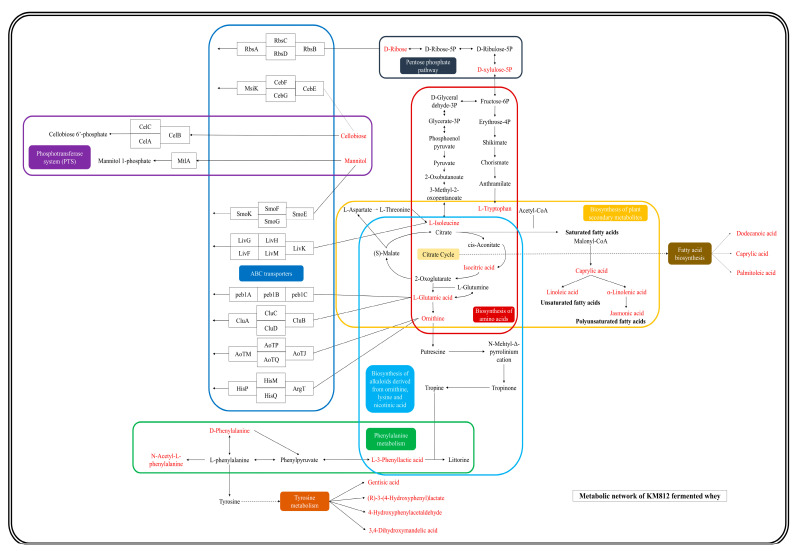
Metabolic network diagram between specific metabolites and KEGG pathways (red markers represent specific metabolites, different coloured rounded rectangles represent different pathways enriched by KEGG analysis).

Enrichment analysis utilizing the KEGG database was employed to associate differential metabolites with specific metabolic pathways, identifying significant pathways and assessing their dynamic changes during fermentation. As depicted in Figure 6, the analysis revealed that differential metabolites predominantly influenced nine pathways, encompassing a total of 23 distinct metabolites. Subsequently, leveraging these metabolite-pathway associations, a comprehensive metabolic pathway map was constructed (Figure 7). Numerous differential metabolites affected by *K. marxianus* KM812 fermentation were identified to participate in ABC transport, as well as amino acid and lipid metabolism pathways. As illustrated in Figure 7, 13 metabolites, such as L-glutamic acid, L-isoleucine, ornithine, cellobiose, mannitol, and D-ribose, were associated with the ABC transport pathway. Metabolites involved in amino acid metabolism, including isocitric acid, L-isoleucine, L-glutamate, and ornithine, were closely linked to the citric acid cycle and various metabolic pathways.

L-3-Phenyllactic acid and D-phenylalanine interconvert within the phenylalanine metabolism pathway, yielding N-acetyl-L-phenylalanine as the final metabolite. D-phenylalanine undergoes conversion into gentisic acid, 4-hydroxyphenylacetaldehyde, (R)-3-(4-hydroxyphenyl)lactate, and 3,4-dihydroxymandelic acid via the tyrosine metabolic pathway. Lipid metabolism pathways primarily involve the transformation of saturated fatty acid octanoic acid into unsaturated fatty acids such as linoleic acid and α-linolenic acid, with the latter further converting to the polyunsaturated fatty acid jasmonic acid. The citrate cycle indirectly influences the fatty acid biosynthesis pathway, involving metabolites such as dodecanoic acid, caprylic acid, and palmitic acid. In conclusion, the fermentation of *K. marxianus* KM812 enhances the bioactive compounds in fermented whey, optimizes nutrient proportions, and increases whey bioavailability.

## 4. Discussion

Fermentation processes possess the capability to degrade macromolecules and modify specific functional factors. By fermenting whey, it facilitates the enrichment of small molecules, thereby enhancing the nutritional and functional properties of whey, promoting improved digestion and absorption efficiency. This study employed LC-MS techniques to investigate the metabolic profile of KM812-fermented whey. The results revealed dynamic changes in the metabolites of KM812-fermented whey over time. A total of 151 differential metabolites were identified, primarily classified into four categories: amino acids, peptides, and analogues; fatty acids and conjugates; carbohydrates and conjugates; and benzoic acids and derivatives. Several of these metabolites significantly augmented the nutritional and functional attributes of KM812-fermented whey.

KM812 has some protein degradation capacity to produce amino acids and analogues (protein hydrolysis products) during the fermentation of whey [8]. This process usually involves the production of proteases by yeast to degrade proteins in whey, which releases amino acids and small molecule polypeptides. Notably, there was a 2-fold increase in L-isoleucine and a 6.38-fold increase in ornithine, both essential amino acids crucial for protein synthesis and various cellular functions. These amino acids were essential for the synthesis of enzymes, structural proteins, hormones, and other vital biomolecules. L-isoleucine could be oxidized to produce energy. During metabolism, it could be converted to pyruvate, which is further involved in the tricarboxylic acid cycle to produce energy molecules such as ATP. Its concentration could act as a signal transducer within the cell, affecting the regulation of protein synthesis and metabolic pathways [21]. Ornithine is a precursor of 5-hydroxytryptophan, an important biological neurotransmitter. Through a series of biochemical reactions, ornithine could be converted to 5-hydroxytryptophan, which is then further synthesized into serotonin (5-hydroxytryptophan) and melatonin, which play a key role in mood regulation and sleep modulation. And it is also involved in the biosynthesis of the coenzyme NAD+, an important coenzyme required for many metabolic reactions [22].

Betaine was detected in KM812-fermented whey, a stable and non-toxic natural product known to prevent alcohol-induced hepatic steatosis, apoptosis, and the accumulation of damaged proteins. Furthermore, it significantly attenuates progressive liver injury by preserving intestinal integrity and adipose function [23]. During the fermentation of whey with KM812, betaine exhibited an increasing trend, peaking at 72 h with a 2.41-fold increase in content. Additionally, L-theanine, a non-protein amino acid with notable biological activities including anti-cerebral ischemia–reperfusion injury, decompression, anti-tumor, anti-ageing, and anti-anxiety effects [24], also showed a 2.19-fold increase in content during the fermentation of whey with KM812.

Another significant finding in this study was a 20.81-fold increase in the α-linolenic acid content during the fermentation of whey with KM812. α-linolenic acid serves as the precursor for three crucial longer-chain n-3 fatty acids: eicosapentaenoic acid (EPA 20:5ω3), docosapentaenoic acid (DPAω3 22:5ω3), and docosahexaenoic acid (DHA 22:6ω3). These acids play pivotal roles in brain development and function, cardiovascular health, and the inflammatory response [25]. Additionally, palmitoleic acid, which saw a 9.6-fold increase during fermentation, possesses physiological functionalities that contribute to weight control, type II diabetes prevention, cardiovascular disease prevention, and anti-inflammatory properties [26]. Thus, KM812 fermentation effectively enhances the functional properties of whey and increases the bioavailability of lipids.

In conclusion, KM812 fermentation significantly enhanced the nutritional quality, functional properties, and bioavailability of whey. Moreover, investigating the dynamic changes in various functional factors can establish a theoretical foundation for the development of functional foods. Nonetheless, this study has its limitations, as the initial metabolic profiles do not fully elucidate the mechanisms underlying the synthesis or degradation of different metabolites. Future research should incorporate additional omics techniques to analyze the characteristics of the microbial proteome.

## 5. Conclusions

This study employed an untargeted metabolomics approach based on LC-MS to dynamically monitor key metabolites during the fermentation of whey by *Kluyveromyces marxianus* KM812. The metabolic profile of KM812-fermented whey exhibited distinct temporal trends. A total of 151 differential metabolites were identified, categorized into four main groups: amino acids, peptides, and analogues; fatty acids and conjugates; carbohydrates and carbohydrate conjugates; and benzoic acids and derivatives. The fluctuations observed in these primary metabolites were pivotal for shaping the nutritional and functional attributes of KM812-fermented whey. Furthermore, KEGG analysis unveiled the involvement of nine pathways in these metabolic alterations, predominantly associated with amino acid and lipid metabolism.

Through in-depth exploration of metabolite types, mechanisms of action, metabolic pathways and fermentation processes, on the basis of interdisciplinary cooperation and continuous innovation, committed to product development, process optimization, mechanism understanding, and marketing efforts in many aspects, we aim to develop health products suitable for different population needs, and guide people to enjoy the health benefits of these products through scientific diets. Thus, the research results will be transformed into actual productivity and make more contributions to the cause of human health.

## Figures and Tables

**Table 1:** Cluster differential metabolite statistics.

Cluster 1	Cluster 2	Cluster 3	Cluster 4	Cluster 5	Cluster 6	Cluster 7	Cluster 8	Cluster 9
Ketoleucine	Triethylamine	Glyceric acid	1,3-Dihydro-(2H)-indol-2-one	Benzamide	Cytosine	2-Phenylethanol	Isovaleric acid	Antiarol
1-Pyrroline-2-carboxylic acid	4-Hydroxyphenylacetaldehyde	m-Cresol	L-Glutamic acid	L-Carnitine	Isonicotinic acid	2,6-Dimethylaniline	trans-trans-Muconic acid	Gamma-Glutamyl-beta-aminopropiononitrile
Ornithine	Gentisic acid	L-Iditol	(3S)-6-Acetamido-3-aminohexanoate	(2S,5S)-trans-Carboxymethylproline	Chavicol	L-Theanine	Decanoyl-L-carnitine	N-Acetyl-D-galactosamine
2-Naphthol	Miglitol	Anserine	Jasmonic acid	(R)-3-(4-Hydroxyphenyl)lactate	Aminoadipic acid	Glucosamine	L-Ribulose	3-Dehydrosphinganine
Biotin	Cytidine	Arachidic acid	Dodecanoic acid	3,4-Dihydroxymandelic acid	Spermidine	Kynurenic acid	10-Hydroxydecanoic acid	1-O-Feruloyl-beta-D-glucose
N6-(L-1,3-Dicarboxypropyl)-L-lysine	Glycerophosphocholine	Labetalol	4-Hydroxycinnamoylagmatine	N-Acetylhistidine	Nicotine	Lumichrome	5-Hydroxyindoleacetic acid	Caprylic acid
Gamma-Glutamyltyramine	(5-L-Glutamyl)-L-glutamate	Docosapentaenoic acid (22n-3)	Methylmalonic acid	Dibutyl phthalate	L-Methionine S-oxide	Galactosylglycerol	L-Cystine	Threonic acid
gamma-L-Glutamyl-L-cysteinyl-beta-alanine	Octadecanamide		Picolinic acid	Palustradienal	beta-Selinene	epsilon-(gamma-L-Glutamyl)-L-lysine		Tropate
2-Hydroxy-6-pentadecylbenzoic acid	N-Acetyl-a-neuraminic acid		2-Pyrocatechuic acid	Androstenedione	L-Kynurenine	Triethyl citrate		beta-D-Glucosamine
Nobiletin	Oleoylethanolamide		Phenylacetylglycine	Sphinganine	Pantetheine	Cyclopeptine		Formylanthranilic acid
Glycochenodeoxycholic acid	dAMP		Phenylacetylglutamine	8(R)-Hydroperoxylinoleic acid	3-Ketosphingosine	Linoleic acid		L-3-Phenyllactic acid
Retinoyl b-glucuronide	L-Tryptophan		all-trans-Retinoic acid	beta-Lactose	D-4’-Phosphopantothenate	2-Hydroxyestrone		N-Acetylleucine
D-Arabitol	N-Acetyl-L-phenylalanine			5alpha-Cholestanone	CMP	L-Octanoylcarnitine		Myristoleic acid
Suberic acid	17a-Estradiol			alpha-Tocopherol	Tangeritin	17-Hydroxyprogesterone		16-Hydroxy hexadecanoic acid
Deoxyguanosine	Alpha-Linolenic acid			26-Hydroxyecdysone	Rutin	Lubiprostone		19(S)-HETE
[8]-Shogaol	Cellobiose			alpha-Ketoisovaleric acid	O-Phosphoethanolamine	Genistin		Arachidonic acid
N-Acetylmuramate	Riboflavin			Betaine	2-Carboxybenzaldehyde	L-Isoleucine		9,10-DHOME
				Beta-Leucine	Glucuronic acid-3,6-lactone	D-Phenylalanine		Fructose 1,6-bisphosphate
				D-Ribose	Isocitric acid	Carglumic acid		
				1H-Indole-3-carboxaldehyde	Nonadecanoic acid	Palmitoleic acid		
				Mannitol	N-Acetyl-D-Glucosamine 6-Phosphate	Apigenin		
				Gluconic acid	Cyclic AMP	dTMP		
				Thymidine	ATP			
				Xylulose 5-phosphate				
				Xylitol 5-phosphate				
				2-Keto-3-deoxy-6-phosphogluconic acid				
				dGMP				
				Glycocholic acid				

## Data Availability

All data generated or analyzed during this study are included in this published article and its Supplementary Information Files.

## Supplementary Materials

The following supporting information can be downloaded at https://www.mdpi.com/article/10.3390/metabo14120694/s1. Table S1: Differential metabolite identification quantification scale.
