# Supplementary material for: Non-Targeted Metabolomics Analysis Reveals Metabolite Profiles Change During Whey Fermentation with Kluyveromyces marxianus
Source: Metabolites. 2024 Dec 9;14(12):694. doi: 10.3390/metabo14120694 (PMC11676789; doi:10.3390/metabo14120694)
Supplement: Supplementary file 1 [file metabolites-14-00694-s001.zip › metabolites-3301591-supplementary.pdf]

Table S1. Differential metabolite identification quantification scale

Metabolites	m/z	Retention time (min)	Compound ID	Class	Sub-class	Relative Abundance			
						0h	24h	48h	72h
(5-L-Glutamyl)-L-glutamate	259.0924	162	M259T162	Carboxylic acids and derivatives	Amino acids, peptides, and analogues	706179.1747	58001237.15	63542447.76	65847074.2
2-Keto-3-deoxy-6-phosphogluconic acid	258.007	329.1	M258T329_1	Carboxylic acids and derivatives	Alcohols and polyols	43184358.56	61420174.74	75151283.53	79963796.97
8(R)-Hydroperoxylinoleic acid	313.2373	734	M313T734	Carboxylic acids and derivatives	Alkyl hydroperoxides	23497264.8	31898935.75	37003675.76	34532510.04
Aminoadipic acid	144.0653	294.2	M144T294	Carboxylic acids and derivatives	Amino acids, peptides, and analogues	238021171.5	260337496.1	272610105.5	263947565.3
Betaine	116.071	923.4	M116T923	Carboxylic acids and derivatives	Amino acids, peptides, and analogues	13014185.18	19569064.81	29769979.35	31380176.63
Beta-Leucine	130.0881	672.5	M130T673	Carboxylic acids and derivatives	Amino acids, peptides, and analogues	65579343.57	109917011.3	134566644.4	137210398.8
Carglumic acid	189.057	407.5	M189T407_1	Carboxylic acids and derivatives	Amino acids, peptides, and analogues	1337827.146	13106420.38	9236764.567	10650626.44
D-4'-Phosphopantothenate	300.0853	224.9	M300T225	Carboxylic acids and derivatives	Alcohols and polyols	1701328.699	68380218.5	76787278.72	36620639.95
gamma-Glutamyl-beta-aminopropiononitrile	199.1073	307.9	M199T308	Carboxylic acids and derivatives	Amino acids, peptides, and analogues	720223560.4	473320057.8	506119424.3	430903608.4
Isocitric acid	191.0195	376.2	M191T376	Carboxylic acids and derivatives	Alcohols and polyols	23482962.91	31708134.59	39504460.73	34279820
L-Cystine	239.1281	481.1	M239T481	Carboxylic acids and derivatives	Amino acids, peptides, and analogues	40758248.07	42663131.89	40846932.27	19211268.27
L-Glutamic acid	148.0608	96.9	M148T97	Carboxylic acids and derivatives	Amino acids, peptides, and analogues	230262410.1	173444917.5	196619804.6	82591613.63
L-Isoleucine	130.0862	540.8	M130T541	Carboxylic acids and derivatives	Amino acids, peptides, and analogues	31223249.05	65691762.96	62366839.82	62954276.78
L-Theanine	157.0972	314	M157T314	Carboxylic acids and derivatives	Amines	43616202.56	102759261.9	95240737.72	95556993.89
Methylmalonic acid	117.0186	917.4	M117T917	Carboxylic acids and derivatives	Dicaboxylic acids and derivatives	37442830.75	28834261.43	17495061.39	17155702.91
N6-(L-1,3-Dicarboxypropyl)-L-lysine	259.1289	122.4	M259T122	Carboxylic acids and derivatives	Amino acids, peptides, and analogues	47563522.81	206762485.9	434760266.4	613000625.1
N-Acetyl-D-Glucosamine 6-Phosphate	300.0483	376.2	M300T376	Carboxylic acids and derivatives	Alcohols and polyols	1035273.129	32657357.18	35167288.7	19680421.91
N-Acetylhistidine	198.0871	107.7	M198T108	Carboxylic acids and derivatives	Amino acids, peptides, and analogues	217346198.6	300482599.5	423400461.5	407726793.4
N-Acetylleucine	172.0967	326.3	M172T326	Carboxylic acids and derivatives	Amino acids, peptides, and analogues	97148042.96	62026310	62887317.68	59511956.29
N-Acetyl-L-phenylalanine	206.0821	356.6	M206T357	Carboxylic acids and derivatives	Amino acids, peptides, and analogues	36643657.42	49086109.5	52998033.52	55035243.09
Ornithine	133.0976	83.5	M133T83	Carboxylic acids and derivatives	Amino acids, peptides, and analogues	25241959.97	37752269.28	120281326	161080269.2

Pantetheine	261.1282	284	M261T284_1	Carboxylic acids and derivatives	Alcohols and polyols	3534867.774	27625045.9	30172437.01	22561359.4
Phenylacetylglutamine	263.1034	340.5	M263T341	Carboxylic acids and derivatives	Amino acids, peptides, and analogues	17920721.73	13437548.27	13687357.36	10347228.06
Phenylacetylglycine	192.1377	348.4	M192T348_3	Carboxylic acids and derivatives	Amino acids, peptides, and analogues	10585934.37	9882342.507	8795476.587	6754717.844
Triethyl citrate	277.1286	654.9	M277T655	Carboxylic acids and derivatives	Tricarboxylic acids and derivatives	629906.5598	12278828.13	12489905.37	11709829.56
16-Hydroxy hexadecanoic acid	271.2276	828	M271T828	Fatty Acyls	Fatty acids and conjugates	636773122.7	310550771.6	406303247.5	279557641.9
19(S)-HETE	301.2162	871.3	M301T871	Fatty Acyls	Eicosanoids	42790237.07	28819171.88	25628020.06	25345574.26
9,10-DHOME	313.2386	711.5	M313T711_2	Fatty Acyls	Fatty acids and conjugates	124995731.5	113423080.5	108361254.8	111568371.5
Alpha-Linolenic acid	278.2199	882.3	M278T882	Fatty Acyls	Lineolic acids and derivataives	3024733.412	44977107.88	54174789.63	62945831.7
Arachidic acid	311.2948	925.8	M311T926	Fatty Acyls	Fatty acids and conjugates	18919055.1	18570692.92	20216910.26	29693583.5
Arachidonic acid	303.2325	897.8	M303T898	Fatty Acyls	Fatty acids and conjugates	3297170.492	2195455.466	2402764.358	1870305.102
Caprylic acid	124.9911	108.6	M125T109	Fatty Acyls	Fatty acids and conjugates	32144907.04	29996261.18	29882765.48	29258278.84
Docosapentaenoic acid (22n-3)	329.2483	916.1	M329T916	Fatty Acyls	Fatty acids and conjugates	48654261.42	47953435.47	48616298.61	57290569.06
Dodecanoic acid	199.9885	970	M200T970	Fatty Acyls	Fatty acids and conjugates	61691455.32	45096237.23	28624464.54	13352647.13
Isovaleric acid	102.1281	210.5	M102T211	Fatty Acyls	Fatty acids and conjugates	101644819.5	115735795.4	98046482	53274606.16
Jasmonic acid	193.1228	780	M193T780	Fatty Acyls	Lineolic acids and derivataives	40834349.72	31056629.55	30512996.81	22249109.47
Linoleic acid	281.2479	826.9	M281T827	Fatty Acyls	Lineolic acids and derivataives	17712624.47	59504031.13	52194176.78	46526350.36
L-Octanoylcarnitine	288.2172	562.2	M288T562	Fatty Acyls	Fatty acid esters	25014415.28	267031516.5	269992305	237590314.4
Lubiprostone	391.2244	865.3	M391T865	Fatty Acyls	Eicosanoids	766688.7859	19729722.04	17949727.31	16430180.02
Myristoleic acid	226.1889	849.1	M226T849	Fatty Acyls	Fatty acids and conjugates	22433948.81	11643313.45	15306816.99	12009273.79
Nonadecanoic acid	297.2437	862.4	M297T862	Fatty Acyls	Fatty acids and conjugates	71383224.31	218248397.3	273217149.1	189809105.6
Octadecanamide	284.2952	826.9	M284T827	Fatty Acyls	Fatty amides	83698187.7	499624779.6	573359665.4	593253053.7
Palmitoleic acid	254.2207	911.4	M254T911	Fatty Acyls	Fatty acids and conjugates	9288069.9	89215232.45	75036452.26	63158237.46
Suberic acid	173.0816	310.2	M173T310	Fatty Acyls	Fatty acids and conjugates	18286929.53	51394259.27	71753192.64	92404178.35
trans-trans-Muconic acid	143.0343	281.9	M143T282	Fatty Acyls	Fatty acids and conjugates	215669537.4	275961994.4	115266276.1	86484987.42

3-Dehydrosphinganine	300.2901	719	M300T719	Organooxygen compounds	Carbonyl compounds	16791838.14	15090051.25	15072604.5	14544979.03
D-Arabitol	151.0604	112.4	M151T112	Organooxygen compounds	Carbohydrates and carbohydrate conjugates	32180335.59	171092951.7	320648514	471671758.4
D-Ribose	131.0343	100.4	M131T100	Organooxygen compounds	Carbohydrates and carbohydrate conjugates	323864459.1	851036327	2028997128	1557212023
Fructose 1,6-bisphosphate	339.1993	828.6	M339T829	Organooxygen compounds	Carbohydrates and carbohydrate conjugates	96127504.54	84741589.3	86322976.26	82719144.05
Gluconic acid	195.0506	89	M195T89	Organooxygen compounds	Carbohydrates and carbohydrate conjugates	205890193.3	220827707.5	246578956.1	244966267.4
Glucosamine	162.0763	176.5	M162T176	Organooxygen compounds	Carbohydrates and carbohydrate conjugates	19490451.16	192046223.3	134332243.1	93062019.08
Glyceric acid	105.0191	90	M105T90	Organooxygen compounds	Carbohydrates and carbohydrate conjugates	170492366.7	147408196.4	168214107.6	340665078.1
L-Iditol	181.0704	588.6	M181T589	Organooxygen compounds	Carbohydrates and carbohydrate conjugates	30272424.05	39478722.96	40110528.13	72969889.48
L-Kynurenine	209.0922	322.1	M209T322	Organooxygen compounds	Carbonyl compounds	9743340.233	108257980.5	110565102.9	68861800.55
L-Ribulose	150.0552	242.2	M150T242	Organooxygen compounds	Carbohydrates and carbohydrate conjugates	116014317.8	137920007.2	120633859.4	66854763.01
Mannitol	181.0714	98.3	M181T98	Organooxygen compounds	Carbohydrates and carbohydrate conjugates	254294490.1	628079347.6	1187285504	1136884777
N-Acetyl-a-neuraminic acid	292.1025	100.4	M292T100	Organooxygen compounds	Carbohydrates and carbohydrate conjugates	155807860.4	451256736.8	423964736.7	597502942
N-Acetylmuramate	292.1041	105.2	M292T105	Organooxygen compounds	Carbohydrates and carbohydrate conjugates	197003547	461307464	1056077227	1789381452
Threonic acid	135.0296	89.5	M135T90	Organooxygen compounds	Carbohydrates and carbohydrate conjugates	355299215.5	232894940.7	195461414.5	224169194.8
beta-Lactose	343.1241	144.9	M343T145	Carbohydrates and carbohydrate conjugates	Glycosyl compounds	13121239.95	368789928.2	626687247.8	722827602.9
(R)-3-(4-Hydroxyphenyl)lactate	183.0653	66.8	M183T67	Benzene and substituted derivatives	1-hydroxy-2-unsubstituted benzenoids	48398464.09	99161345.74	129196091.2	119331800.9
[8]-Shogaol	275.1679	709.5	M275T709	Benzene and substituted derivatives	Phenols and derivatives	6998676.378	26470105.92	57054420.32	90302568.51
2,6-Dimethylaniline	122.0969	358.8	M122T359	Benzene and substituted derivatives	Xylenes	11170789.54	35490391.78	32269069.88	28746430.78
2-Carboxybenzaldehyde	149.0246	445.7	M149T446	Benzene and substituted derivatives	Benzoic acids and derivatives	2777111.185	16294724.2	23620616.26	12683437.07
2-Hydroxy-6-pentadecylbenzoic acid	348.2749	632.8	M348T633	Benzene and substituted derivatives	Benzoic acids and derivatives	4162218.791	15123587.31	34049200.95	59536049.84
2-Phenylethanol	105.0705	358.8	M105T359	Benzene and substituted derivatives		8755385.331	31146560.79	29009760.17	28650498.54
2-Pyrocatechuic acid	153.0185	410.2	M153T410	Benzene and substituted derivatives	Benzoic acids and derivatives	124273962.3	107046945.1	79223001.1	69316880.57
4-Hydroxyphenylacetaldehyde	137.0626	247.8	M137T248	Benzene and substituted derivatives	Phenylacetaldehydes	7659207.17	33512619.1	31679773.27	35618485.29
Benzamide	122.0602	442.5	M122T442	Benzene and substituted derivatives	Benzoic acids and derivatives	44911147.19	75688909.5	98822635.64	98090281.07

Dibutyl phthalate	279.1597	841.4	M279T841	Benzene and substituted derivatives	Benzoic acids and derivatives	1856588.184	65306283.52	98965555.5	100018667.2
Formylanthranilic acid	164.0351	331.8	M164T332	Benzene and substituted derivatives	Benzoic acids and derivatives	64587024.41	12543281.41	16004306.69	11550709.54
Gentisic acid	154.9906	972.6	M155T973	Benzene and substituted derivatives	Benzoic acids and derivatives	9707505918	11422885926	11859323128	12312148199
Labetalol	328.1831	743.6	M328T744	Benzene and substituted derivatives	Benzoic acids and derivatives	53897648.85	40500173.29	58434726.98	70970743
CMP	324.0593	121.6	M324T122	Pyrimidine nuceotides	Pyrimidine ribonucleotides	5099688.749	76052704.49	82760524.94	67353578.44
dTMP	321.0489	144.7	M321T145	Pyrimidine nuceotides	Pyrimidine deoxyribonucleotides	48845.43148	210972.9797	196631.7284	164394.649
Thymidine	223.028	708.6	M223T709_1	Pyrimidine nucleosides	Pyrimidine 2'-deoxyribonucleotides	13323347.91	36643279.11	48122228.03	52470629.23
ATP	507.1175	881.8	M507T882	Purine nucleotides	Purine ribonucleotides	1969915.603	13635473.49	18228940.32	14342206.51
Cyclic AMP	328.0449	295.9	M328T296	Purine nucleotides	Cyclic purine nucleotides	9666147.958	200704939.5	232979945.5	180042817.7
dAMP	332.0752	158.6	M332T159_1	Purine nucleotides	Purine deoxyribonucleotides	22510794.12	90586860.62	103622044	103817559.3
dGMP	346.0547	152.2	M346T152	Purine nucleotides	Purine deoxyribonucleotides	8466943.208	91417587.62	121818782.6	133273558.5
Deoxyguanosine	266.0843	243.8	M266T244	Purine nucleosides	Purine 2'-deoxyribonucleotides	4287641.973	15852475.86	21368749.33	31422176.25
beta-D-Glucosamine	160.0614	815.1	M160T815	Organonitrogen compounds	Alcohols and polyols	192743069.4	118701715.6	101574217.8	76350356.59
L-Carnitine	162.1128	391.6	M162T392	Organonitrogen compounds	Quaternary ammonium salts	57030257.25	99869745.97	130600801.4	114733924.6
Oleoylethanolamide	326.3057	917	M326T917	Organonitrogen compounds	Amines	3902069.329	25403697.89	29806214.81	36617951.04
Spermidine	146.1656	76.7	M146T77	Organonitrogen compounds	Amines	199766554.7	946862762.7	1007933247	711440176.3
Sphinganine	302.4438	739.5	M302T739	Organonitrogen compounds	Amines	265512.1731	8596560.94	12053397.71	12516864.25
Triethylamine	101.0604	46.8	M101T47	Amines	Tertiary amines	1488220.575	18065162.03	17511471.22	18616704.09
Palustradienal	286.2381	833.8	M286T834	Organic oxides	Carbonyl compounds	11369603.59	274339750.1	252632791.7	184853298.7
2-Hydroxyestrone	286.1496	154.2	M286T154	Organic oxides	1-hudroxy-2-unsubstituted benzenoids	23595.33742	13583567.5	23930447.15	27595441.45
Androstenedione	287.1969	660.6	M287T661	Organic oxides	Androstane steroids	1131682.008	10041202.1	14821241.36	14028773.5
Xylitol 5-phosphate	231.0271	117.3	M231T117	Organic oxides	Alcohols and polyols	1213019.377	1445147.835	2890691.084	2862719.965
Xylulose 5-phosphate	230.0124	307.5	M230T308	Organic oxides	Alcohols and polyols	3708920.028	5392048.969	6616930.322	6503865.328
1,3-Dihydro-(2H)-indol-2-one	134.0604	493.4	M134T493	Indoles and derivatives	Indolines	30098431.19	26747525.81	26301269.52	21940915.95

1H-Indole-3-carboxaldehyde	144.0457	541.9	M144T542	Indoles and derivatives	Indoles	31081740.8	41395702.28	87531934.7	70190654.41
5-Hydroxyindoleacetic acid	191.1081	740.2	M191T740	Indoles and derivatives	Indolyl carboxylic acids and derivtives	12737672.17	16425181.32	9409177.791	2371276.574
L-Tryptophan	203.0822	343.5	M203T343	Indoles and derivatives	Indolyl carboxylic acids and derivtives	7925089.961	18923169.51	16204355.17	20601269.61
all-trans-Retinoic acid	299.2012	856.8	M299T857	Prenol lipids	Retinoids	11147293.35	9626197.635	6731705.62	3395791.617
alpha-Tocopherol	430.2432	488.6	M430T489	Prenol lipids	Quinone and hydroquinone lipids	2064953.654	28808296.62	44157632.09	51882262.54
beta-Selinene	205.1956	850.6	M205T851	Prenol lipids	Sesquiterpenoids	1706094.5	10518316.89	13031593.49	8648170.28
Retinoyl b-glucuronide	476.2769	745.6	M476T746	Prenol lipids	Terpene glycosides	2093378.022	11458497.52	21325292.1	29861475.41
Apigenin	270.0471	293.4	M270T293	Flavonoids	Flavones	341154.3398	8122304.356	6735204.145	5344246.552
Nobiletin	403.1383	724.5	M403T724	Flavonoids	O-methylated flavonoids	136519.91	1586026.793	2074099.405	5165227.65
Tangeritin	373.1295	750.1	M373T750	Flavonoids	O-methylated flavonoids	73215.49371	3567352.588	4496806.709	3582013.138
Genistin	432.2369	754.7	M432T755_1	Isoflavonoids	Isoflavonoid O-glycosides	9371905.75	487641573.3	516610586.7	443050034.8
17a-Estradiol	271.2276	792.8	M271T793_2	Steroids and steroid derivatives	Estrane steroids	620907082	2505735996	2942643577	2917280264
17-Hydroxyprogesterone	331.2303	767.5	M331T768	Steroids and steroid derivatives	Pregnane steroids	1098679.957	19003030.21	20223986.99	18176526.26
5alpha-Cholestanone	369.3532	891.6	M369T892	Steroids and steroid derivatives	Cholestane steroids	3723039.53	80109606.27	117495026.6	147812773.5
3,4-Dihydroxymandelic acid	185.0427	86.2	M185T86_1	Phenols	Benzenediols	39490027.5	249156898.8	383463873.1	376900545.4
Chavicol	135.0807	581.8	M135T582	Phenols	1-hydroxy-2-unsubstituted benzenoids	11148514.86	16967508.63	19443238.91	16889382.08
m-Cresol	107.0495	438	M107T438	Phenols	Cresols	110151990.3	91608302.37	108446764	219472975.8
Isonicotinic acid	124.0396	440.3	M124T440	Pyridines and derivatives	Pyridinecarboxylic acids and derivatives	34674865.14	180776769.4	200032086	157676687.5
Nicotine	163.1234	159.6	M163T160	Pyridines and derivatives	Pyrrolidinylpyridines	36123597.66	64846092.28	73164273.65	63695556.26
Picolinic acid	122.0239	749.5	M122T749	Pyridines and derivatives	Pyridinecarboxylic acids and derivatives	50807779.43	33942642.64	25347632.3	16623825.45
L-Methionine S-oxide	166.0532	156.2	M166T156	Endogenous Metabolites		263215307.1	423280667.7	603673841.4	452183193.3
N-Acetyl-D-galactosamine	204.0852	391.9	M204T392	Endogenous Metabolites		285239160.2	213981224.7	212402115.8	190320155.3
Glucuronic acid-3,6-lactone	157.0115	112.7	M157T113	Endogenous Metabolites; Excipients/Additives/Colorants		37783301.23	171662966.7	218543524.7	134922707
alpha-Ketoisovaleric acid	115.0397	161.8	M115T162	Keto acids and derivatives	Short-chain keto acids and derivatives	4649905.601	7706972.834	10718406.91	10915186.72

Ketoleucine	113.0349	157.1	M113T157	Keto acids and derivatives	Short-chain keto acids and derivatives	46104048.26	56967528.44	58408229.72	66939561.9
1-Pyrroline-2-carboxylic acid	114.0661	532.6	M114T533	Pyrrolines		1321871492	3397499143	8187286665	9513395196
2-Naphthol	144.0668	988.3	M144T988	Naphthalenes	Naphthols and derivatives	142883507	147152893.2	164742800.3	169545828.9
4-Hydroxycinnamoylagmatine	276.1447	265.7	M276T266	Cinnamic acids and derivatives	Hydroxycinnamic acids and derivatives	540506164.8	433244082.1	394889660.8	345335949.1
Anserine	239.1143	109.1	M239T109	Peptidomimetics	Hybrid peptides	5083571.586	5021134.434	5485756.27	8986729.893
Biotin	245.0957	431.7	M245T432	Biotin and derivatives		33841496.41	41105500.24	50585454.7	57984563.39
Cytosine	112.0512	108.2	M112T108_1	Diazines	Pyrimidines and pyrimidine derivatives	77858788.16	376875586.3	436611727.7	280853473
Galactosylglycerol	255.1058	104.8	M255T105	Glycerolipids	Alcohols and polyols	83542602.06	2986412423	2163183807	2167106801
Glycerophosphocholine	258.1105	146.6	M258T147_1	Glycerophospholipids	Glycerophosphocholines	62662795.29	322942382.3	259306443.5	324937246.4
Kynurenic acid	190.05	391.5	M190T391	Quinolines and derivatives	Quinoline carboxylic acids	24127901.05	100203450.1	101515849	88898500.3
L-3-Phenyllactic acid	166.0588	331.5	M166T331	Phenypropanoic acids		60582574.79	46474336.38	44112584.46	47191255.97
Miglitol	190.107	242.2	M190T242	Piperidines		30888297.58	90454609.52	101184816.4	125405297.4
O-Phosphoethanolamine	140.0116	87.1	M140T87	Organic phosphoric acids and derivatives	Phosphate esters	71310799.65	172405303.4	211877793.2	181469615.5
Riboflavin	375.1286	411.5	M375T412	Pteridines and derivatives	Alloxazines and isoalloxazines	3718968.068	38056202.37	33406662.53	57522116.77
Tropate	147.0448	331.5	M147T331	Hydroxy acids and derivatives	Beta hydroxy acids and derivatives	66565475	35137276.9	43793436.65	38671610.26
(2S,5S)-trans-Carboxymethylproline	174.0767	108.3	M174T108_1	Unclassified		86733925.44	160348386.8	224962732.1	229473211.1
(3S)-6-Acetamido-3-aminohexanoate	188.1158	194.2	M188T194	Unclassified		27543479.51	19315652.36	10030717.05	9936326.598
10-Hydroxydecanoic acid	187.133	539.7	M187T540_2	Unclassified		97912194.31	132032760.2	77050366.57	16168261.72
1-O-Feruloyl-beta-D-glucose	339.1125	389	M339T389	Unclassified		59224401.16	21308404.14	23623785.2	14695469.5
26-Hydroxyecdysone	480.2779	593.6	M480T594	Unclassified		5977424.943	18752106.89	33148419.52	32223645.15
3-Ketosphingosine	280.2643	797.7	M280T798	Unclassified		557154.346	18707509.26	24302134.27	20091338.21
Antiarol	167.0709	111.2	M167T111	Unclassified		176534754.2	104453059.2	114113410.8	100282518.6
Cellobiose	341.1075	113.3	M341T113	Unclassified		6288384.955	415415034.3	403659324.6	423955471.1
Cyclopeptine	281.1137	382	M281T382	Unclassified		10369263.52	172583376	144784245.6	117401099.7

Cytidine	244.0928	108.2	M244T108_1	Unclassified	8072615.5	215915345.2	170156483.4	223821371.5
Decanoyl-L-carnitine	316.2488	640.6	M316T641	Unclassified	50268019.56	62344271.6	28242372.33	19339424.94
D-Phenylalanine	164.0711	297.4	M164T297_2	Unclassified	39834860.8	306171566.2	268947702.4	254920133.5
epsilon-(gamma-L-Glutamyl)-L-lysine	276.1633	710.7	M276T711	Unclassified	1171752.737	14159868.49	13296532.24	13680879.12
gamma-Glutamyltyramine	267.1304	283.6	M267T284_1	Unclassified	2265078.195	39043460.6	62996874.77	101446310.2
gamma-L-Glutamyl-L-cysteinyl-beta-alanine	322.0771	216.8	M322T217	Unclassified	219602958.9	879242284.2	1542786858	3091730676
Glycochenodeoxycholic acid	450.3196	724.5	M450T725_1	Unclassified	6339458.409	43740697.49	96754413.04	188059854.5
Glycocholic acid	464.2986	535.4	M464T535_2	Unclassified	20496892.5	207917138.8	342810995.4	387251367.7
Lumichrome	243.0897	522.6	M243T523	Unclassified	39145738.88	604480284.9	565105279.5	487313633.7
Rutin	610.1825	933.7	M610T934	Unclassified	427443.0922	16959445.33	22214618.61	15623793.55

Table S2. KEGG Metabolic Pathway Enrichment Table

Pathway	Hits	Pvalue	FDR	Impact
ABC transporters	13	2.5624E-05	0.0138627389189428	0.0949
Biosynthesis of plant secondary metabolites	11	0.00061248	0.149555136122635	0.078
Biosynthesis of amino acids	10	0.00107285	0.149555136122635	0.0781
Biosynthesis of alkaloids derived from ornithine, lysine and nicotinic acid	7	0.00114472	0.149555136122635	0.1045
Pentose phosphate pathway	5	0.00145647	0.149555136122635	0.1429
Phosphotransferase system (PTS)	6	0.00248798	0.149555136122635	0.1053
Phenylalanine metabolism	6	0.00323304	0.174907241030816	0.1
Tyrosine metabolism	4	0.12236326	0.60661766915765	0.0513
Fatty acid biosynthesis	3	0.1677844	0.703505926855393	0.0517
